# Mechanical force modulates periodontal ligament stem cell characteristics during bone remodelling via TRPV4

**DOI:** 10.1111/cpr.12912

**Published:** 2020-09-22

**Authors:** Shan‐Shan Jin, Dan‐Qing He, Yu Wang, Ting Zhang, Hua‐Jie Yu, Zi‐Xin Li, Li‐Sha Zhu, Yan‐Heng Zhou, Yan Liu

**Affiliations:** ^1^ Laboratory of Biomimetic Nanomaterials Department of Orthodontics Peking University School and Hospital of Stomatology National Engineering Laboratory for Digital and Material Technology of Stomatology Beijing Key Laboratory of Digital Stomatology Beijing China; ^2^ Fourth Division Peking University Hospital of Stomatology Beijing China

## Abstract

**Objectives:**

Mechanical force plays an important role in modulating stem cell fate and behaviours. However, how periodontal ligament stem cells (PDLSCs) perceive mechanical stimulus and transfer it into biological signals, and thereby promote alveolar bone remodelling, is unclear.

**Materials and Methods:**

An animal model of force‐induced tooth movement and a compressive force in vitro was used. After force application, tooth movement distance, mesenchymal stem cell and osteoclast number, and proinflammatory cytokine expression were detected in periodontal tissues. Then, rat primary PDLSCs with or without force loading were isolated, and their stem cell characteristics including clonogenicity, proliferation, multipotent differentiation and immunoregulatory properties were evaluated. Under compressive force in vitro, the effects of the ERK signalling pathway on PDLSC characteristics were evaluated by Western blotting.

**Results:**

Mechanical force in vivo induced PDLSC proliferation, which was accompanied with inflammatory cytokine accumulation, osteoclast differentiation and TRPV4 activation; the force‐stimulated PDLSCs showed greater clonogenicity and proliferation, reduced differentiation ability, improved induction of macrophage migration, osteoclast differentiation and proinflammatory factor expression. The biological changes induced by mechanical force could be partially suppressed by TRPV4 inhibition. Mechanistically, force‐induced activation of TRPV4 in PDLSCs regulated osteoclast differentiation by affecting the RANKL/OPG system via ERK signalling.

**Conclusions:**

Taken together, we show here that TRPV4 activation in PDLSCs under mechanical force contributes to changing their stem cell characteristics and modulates bone remodelling during tooth movement.

## Graphical abstract

Mechanical force in vivo induced periodontal ligament stem cell proliferation, which was accompanied with inflammatory cytokine accumulation, osteoclast differentiation and TRPV4 activation. The activation of TRPV4 in PDLSCs under mechanical force contributes to the changes in their stem cell characteristics including clonogenicity, proliferation, multipotent differentiation, and immunoregulation and modulates bone remodelling during tooth movement.

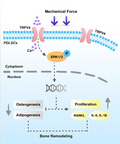

## 1 INTRODUCTION

Mesenchymal stem cells (MSCs) contribute to multiple physiological and pathological processes, including development, inflammation, disease recovery, tissue remodelling and regeneration.^1^, ^2^, ^3^ Mechanical force plays an important role in modulating MSC fate and behaviours, and thereby guiding development, homoeostasis and regeneration.^4^ Notably, existing experiments in vivo have shown that mechanical force influences MSC function during force‐induced bone remodelling or cardiac injury remodelling.^5^, ^6^ Furthermore, mechanical stimuli in vitro—including pressure, shear stress, and stretch might modulate MSC differentiation through mobilization of second messengers, ion channels, cytoskeleton transformation or membrane proteins, such as integrins.^7^

The periodontal ligament (PDL) is a soft connective tissue embedded between two different hard tissues, the root of tooth and the alveolar bone, that stabilizes teeth and relieves the pressure caused by masticatory force.^8^ It plays an important role in maintaining tissue homoeostasis and provides a microenvironment for inflammatory reactions and bone remodelling under mechanical stimulation. Mechanical force‐induced tooth movement through the alveolar bone relies on the PDL and is a unique local aseptic inflammation‐associated process of bone remodelling.^9^ Periodontal ligament stem cells (PDLSCs), as the main MSCs in the PDL microenvironment,^10^ exhibit clonogenicity, proliferation, multipotent differentiation and immunoregulatory properties. They respond to mechanical force by producing high levels of inflammatory cytokines and chemokines, which play vital function in alveolar bone remodelling.^11^, ^12^, ^13^ However, the mechanism by which PDLSCs perceive the mechanical stimulus and transfer it into biological signals, thereby contributing to alveolar bone remodelling, remains to be further investigated.

Transient receptor potential (TRP) calcium channel is a typical mechanosensitive channel and involved in the sensation of different stimuli in various types of tissues and cells.^14^, ^15^ TRP subfamily V member 4 (TRPV4) regulates mechanosensation, inflammation and energy homoeostasis.^16^, ^17^ Moreover, mutations in TRPV4 are associated with inherited disorders of bone metabolism.^18^ MSCs isolated from TRPV4 knockout mice are found to suffer from impaired osteogenic potential.^19^ Therefore, we hypothesized that the stem cell characteristics of PDLSCs are influenced by the activation of TRPV4 under mechanical stimuli, which promotes alveolar bone remodelling and eventually influences tooth movement. Using an animal model of mechanical force‐induced tooth movement and a compressive force stimulus in vitro, we herein evaluated the biological changes in PDLSCs, including their clonogenicity, proliferation, multipotent differentiation and immunoregulatory properties, under mechanical stimuli. Universal upregulation of TRPV4 in PDLSCs and the underlying molecular mechanism under mechanical force are demonstrated. These results together pinpoint the importance of mechanical force‐induced TRPV4 in regulating the stem cell characteristics of PDLSCs and mediating bone remodelling.

## 2 MATERIALS AND METHODS

### 2.1 An experimental animal model of mechanical loading

Male Sprague‐Dawley rats 6‐8 weeks of age (Weitong Lihua Experimental Animal Center, Beijing, China) were used in this study. The animal experimental protocols were approved by the Institutional Animal Care and Use Committee of Peking University (LA2013‐92). To establish an experimental animal model of mechanical loading, nickel‐titanium coil springs (0.2 mm thickness, 1 mm diameter, 4 mm length; Smart Technology) were ligated between the right maxillary first molar and the incisors (Figure S1). Each coil spring can provide a constant force of ~0.6 N to move teeth.^11^, ^20^, ^21^ The contralateral first molar served as the control. After 3 and 7 days of treatment (F 3d, F 7d), the rats (n = 6) were sacrificed by overdose of pentobarbital sodium, and the maxillae were harvested, fixed in 4% paraformaldehyde (PFA) and scanned by micro‐computed tomography (micro‐CT, Skyscan1174, Bruker) at a resolution of 10 μm. The acquired axial images were imported into a NRecon and CTvox software for 3‐D reconstruction. Another 12 rats on day 7 were sacrificed by excessive anaesthesia, and the maxillary first molars of both sides were separated for the culture of primary cells. An occlusal view of the maxillae was obtained using a stereomicroscope (SWZ1000, Nikon). The distance of tooth movement induced by the mechanical loading was measured using a modified method described previously.^21^ Briefly, the distance between two easily located points (the midpoint of the distal‐marginal ridge of the first molar and the midpoint of the mesial‐marginal ridge of the second molar) was measured by two trained researchers, who were blinded to the group assignment. The average of two measurements was calculated as the tooth movement distance.

### 2.2 Haematoxylin and eosin and tartrate‐resistant acid phosphatase staining

The fixed maxillae were demineralized in 15% ethylenediaminetetraacetic acid and embedded in paraffin. Consecutive 4‐μm‐thick transverse sections were obtained from the corresponding group and stained with haematoxylin and eosin (H&E) and tartrate‐resistant acid phosphatase (TRAP) staining. TRAP staining was performed using an acid phosphatase kit (387A‐1KT; Sigma) according to the manufacturer's instructions. TRAP‐positive, multinucleated (>3 nuclei) cells attached to the alveolar bone surface were counted (n = 5).^22^

### 2.3 Immunofluorescence staining

Immunofluorescence staining was performed as described previously.^23^ Tissue sections were double‐stained with anti‐CD146 (1:200, ab‐75769, Abcam) and anti‐Ki67 (1:200, ab8191; Abcam) or anti‐TRPV4 (1:200, ab39260; Abcam) antibodies. Other sections were stained with anti‐interleukin (IL)‐6 (1:200, ab9324; Abcam) and anti‐IL‐1β (1:200, ab9722, Abcam) antibodies. Next, the sections were incubated with fluorescein isothiocyanate‐conjugated or tetramethylrhodamine isothiocyanate‐conjugated secondary antibodies (1:200; Jackson ImmunoResearch Laboratories). Nuclei were counterstained with 4′,6‐diamidino‐2‐phenylindole. Confocal microscopic images were processed with LSM 5 Release 4.2 software following acquisition with a laser‐scanning microscope (LSM 510; Zeiss).

### 2.4 Isolation, culture and treatment of PDLSCs

To obtain rat PDLSCs (rPDLSCs) subjected to in vivo mechanical loading (F‐PDLSCs), periodontal tissues from the rat maxillary force‐induced first molar were slightly separated, minced and digested in a fresh enzyme mixture, comprising 3 mg/mL collagenase type I (Worthington Biochemical) and 4 mg/mL dispase II (Roche Diagnostics) for 60 minutes at 37°C. After passing through a 70‐μm strainer, single‐cell suspensions were cultured in α‐modified Eagle's medium supplemented with 15% foetal bovine serum, 2 mmol/L l‐glutamine and 100 U/mL penicillin/streptomycin. rPDLSCs isolated from normal periodontal tissues (N‐PDLSCs) in the contralateral first molar served as the control. To remove the non‐adherent cells, the cultures were washed twice with phosphate‐buffered saline (PBS). The attached primary cells were cultured for 10 days and then were subcultured. rPDLSCs at passage 2 were used in this study. To inhibit expression of TRPV4 in force‐induced rPDLSCs, a pharmacological antagonist (GSK2193874, Selleck) was added to the culture medium with the final concentration of 10 μmol/L.

Human PDLSCs (hPDLSCs) were isolated from extracted teeth and cultured as described previously.^10^ Permission to obtain extracted teeth was provided by the Ethics Committee of Peking University (PKUSSIRB‐201311103). PDLSCs isolated from 3 different individuals (18‐25 years old) were pooled together, and cells at passages 2‐3 were used in this study. Static compressive force was applied to the hPDLSCs as described previously.^13^ Briefly, a layer of glass and additional metal balls were placed on a 70%‐80% confluent cell layer in six‐well plates. hPDLSCs were subjected to static compressive forces of 0‐1.5 g/cm^2^ for 12 hours.

To inhibit the activation of TRPV4 in hPDLSCs, GSK2193874 (10 μmol/L) was added to the medium for 1 hour prior to compressive force stimulation (1.5 g/cm^2^, 12 hours). To activate the TRPV4, hPDLSCs were treated by a pharmacological agonist (GSK1016790A, Selleck) with the final concentration of 10 nmol/L for 1 hour. For the control group, dimethyl sulfoxide (DMSO) of the same volume was added.

### 2.5 Cell proliferation and colony‐forming units‐fibroblastic assay

Cell proliferation was monitored using a CCK‐8 kit (Sigma) according to the manufacturer's instructions. Rat PDLSCs isolated ex vivo were incubated with CCK‐8 reagent at 37°C for 2 hours. Next, the absorbance at 450 nm was measured using a microplate reader (Bio‐Rad).

Cells (500 per well) were seeded and incubated in six‐well plates for 14 days in growth medium and fixed with 4% PFA (Sigma). Next, 0.1% crystal violet was used to stain the cells. Colonies of more than 50 cells were defined as single colony clusters, and the number of clusters was counted.

### 2.6 Multipotent differentiation of PDLSCs ex vivo

We evaluated the multi‐differentiation potential of rPDLSCs isolated ex vivo with or without force loading towards osteogenesis and adipogenesis as reported previously.^10^ To induce osteogenesis, the medium was changed to osteogenic medium (growth medium with 10 nmol/L dexamethasone, 100 μmol/L ascorbic acid 2‐phosphate and 10 mmol/L β‐glycerophosphate). After culture in osteogenic medium for 21 days, the cells were fixed in 4% PFA and stained with 1% Alizarin Red S (Sigma) at room temperature. The Alizarin Red‐positive area was measured using ImageJ software and expressed as the percentage of Alizarin Red‐positive area over the total area. For adipogenic induction, cells were cultured in adipogenic medium (growth medium with 500 μmol/L isobutyl‐methylxanthine, 60 μmol/L indomethacin, 0.5 μmol/L hydrocortisone and 10 μmol/L insulin) for 3 weeks. After fixing in 60% isopropanol, the cells were stained with 0.3% Oil Red O (Sigma) and the number of Oil Red O‐positive droplet‐containing cells was counted.

### 2.7 Co‐culture of PDLSCs and RAW264.7 macrophages

#### 2.7.1 Transwell migration system

To investigate their chemoattractive activity, rPDLSCs isolated ex vivo with or without force loading were seeded into six‐well plates, and RAW264.7 macrophages were added to the upper chamber of the Transwell migration system at 1 × 10^5^/well. RAW264.7 macrophages were co‐cultured with rPDLSCs isolated ex vivo for 24 hours, and cells remaining in the upper chamber were gently removed using cotton swabs. After washed twice with PBS, the Transwell chambers were fixed in 4% PFA and stained with 0.1% crystal violet for 15 minutes. Cells stained by crystal violet on the bottom surface served as the migrated cells and were counted under an inverted microscope.

#### 2.7.2 Direct cell‐to‐cell contact system

To investigate their osteoclastogenesis, rPDLSCs isolated ex vivo with or without force loading were seeded into 12‐well plates at 5 × 10^3^/well and RAW264.7 macrophages were added at 5 × 10^5^/well. To induce osteoclast differentiation, soluble receptor activator of nuclear factor‐κB ligand (sRANKL) (50 ng/mL) was added to the medium.^24^ After co‐culturing for 7 days, the cells were fixed and stained with an acid phosphatase kit (387A‐1KT; Sigma) for TRAP staining. TRAP‐positive, multinucleated (two or more nuclei) osteoclasts were counted in five visual fields per well (n = 3). The final result was the average of three experiments.

### 2.8 Quantitative reverse transcription polymerase chain reaction (PCR)

Total RNA was extracted using TRIzol reagent (Invitrogen) according to the manufacturer's instructions. Reverse transcription and real‐time PCR were performed following protocols described in detail previously. The primers (designed using Primer Premier 5.0 software) were listed in Table S1. The efficiency of the newly designed primers was confirmed by sequencing the products of conventional PCR.

### 2.9 Western blot analysis

Cells were lysed with RIPA buffer mixed with protease and phosphatase inhibitor cocktail (Thermo Fisher Scientific). Total protein (25 μg) was separated by 10% SDS–polyacrylamide gel and then transferred onto a polyvinylidene difluoride (PVDF) membrane (Millipore). After being blocked in 5% non‐fat milk in Tris‐buffered saline containing 0.1% Tween‐20 for 1 hour at room temperature, the membranes were incubated overnight at 4°C with primary antibodies as following: anti‐TRPV4 (ab39260; Abcam), anti‐Ki67 (ab8191; Abcam), anti‐IL‐6 (ab9324; Abcam), anti‐RANKL (ab45039, Abcam), anti‐osteoprotegerin (OPG) (ab11994, Abcam), anti‐phospho‐p44/42 MAPK (ERK1/2) (Thr202/Tyr204) (4370, Cell Signaling Technology), anti‐p44/42 MAPK (ERK1/2) (4695, Cell Signaling Technology), and anti‐glyceraldehyde‐3‐phosphate dehydrogenase (GAPDH) (3683S, Cell Signaling Technology). Then, the membranes were incubated with a horseradish peroxidase‐conjugated mouse or rabbit IgG (1:5000; Zhongshanjinqiao), and protein bands were detected by enhanced with a SuperSignal West Pico Chemiluminescent Substrate (Thermo Fisher Scientific). The relative density of three comparable results was measured using ImageJ software. Each experiment was repeated three times.

### 2.10 Statistical analysis

Statistical Package for the Social Sciences 19.0 software was used to perform statistical analysis. Data were presented as means ± standard deviation (SD). The normal distribution of the raw data was confirmed by a one‐sample Kolmogorov–Smirnov test, and assessed for significance by two‐tailed independent Student's *t* test or one‐way analysis of variance (ANOVA). Tukey's multiple‐comparison test was used for the post hoc comparison of ANOVA. Differences with *P* < .05 were considered statistically significant.

## 3 RESULTS

### 3.1 Mechanical force induces the proliferation of PDLSCs and the expression of inflammatory factors during bone remodelling and tooth movement

**Figure 1:**
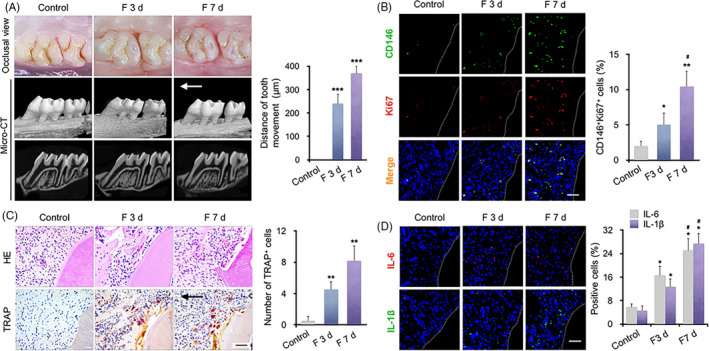
Periodontal ligament stem cells (PDLSCs) and osteoclasts accumulate on the compression side of periodontal tissues following application of mechanical force in vivo. A, Representative occlusal view and micro‐CT images of tooth movement for 3 and 7 d. Semi‐quantitative analysis showed that the distance of tooth movement gradually increased after force was applied for 3 and 7 d (F 3 d and F 7 d, n = 6). The arrow shows the direction of mechanical force. ***P < .001 vs control. B, Representative immunofluorescence images of the compression side of distobuccal roots. The number of CD146^+^ (green) and Ki67^+^ (red) PDLSCs was increased in F 3 d and F 7 d. N = 6, *P < .05, **P < .01, vs control, ^#^ *P* < .05 vs F 3 d. Scale bar: 100 μm. C, Representative H&E and TRAP staining of the compression side of distobuccal roots. The number of TRAP‐positive osteoclasts was increased in the periodontal tissues after force application. The arrow shows the direction of mechanical force. Scale bar: 100 μm. N = 6, **P < .01 vs control. D, Representative immunofluorescence staining and semi‐quantitative analysis of IL‐6 and IL‐1β in the periodontal tissues after force was applied. The number of cells positive for IL‐6 and IL‐1β increased around the periodontal tissues after force was applied. Scale bar: 100 μm. N = 6, *P < .05 vs control. ^#^ *P* < .05 vs F 3 d

The experimental animal model of mechanical loading was fit for purpose, as evidenced by tooth movement of 240.4 ± 40.9 μm on day 3 and 369.7 ± 30.1 μm on day 7 (Figure 1A). Double immunostaining showed that the majority of Ki67‐positive cells (indicating cell proliferation) colocalized with CD146, a surface marker of MSCs, in periodontal tissues. In 3rd day after force application, the proportion of CD146^+^ Ki67^+^ PDLSCs increased to 5.1 ± 1.9% and reached 10.4 ± 2.8% in 7th days, compared with the control group (1.9 ± 0.7%), indicating that the proliferative capacity of PDLSCs enhanced after mechanical force stimulation (Figure 1B). TRAP‐positive osteoclasts accumulated in the periodontal tissues, near the alveolar bone, during mechanical loading (Figure 1C). Moreover, the levels of the proinflammatory cytokines IL‐6 and IL‐1β increased on the compression side of periodontal tissues after mechanical application (Figure 1D). Therefore, mechanical force promotes PDLSC proliferation in vivo, which might account for the accumulation of proinflammatory factors and osteoclast differentiation around the periodontal tissues.

### 3.2 Mechanical force changes the MSC characteristics of rPDLSCs

Although PDLSCs have been reported to possess self‐renewal and multidirectional differentiation potential, their detailed characteristics under mechanical stimuli during bone remodelling remain to be elucidated. Herein, rPDLSCs with or without mechanical loading were isolated and a variety of experimental techniques were applied for their characterization.

**Figure 2:**
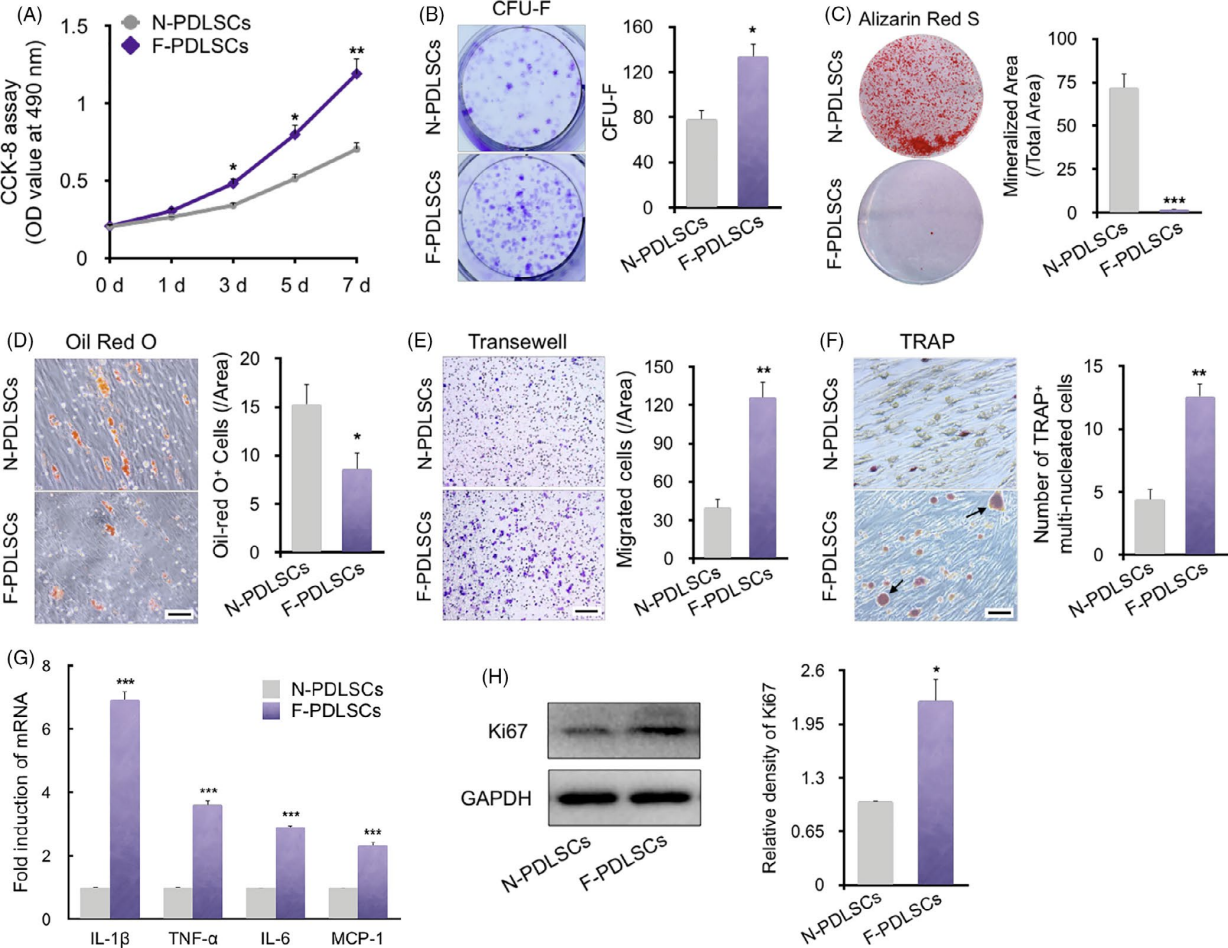
Biological characteristics of force‐induced rPDLSCs ex vivo. A, Growth curves of force‐induced PDLSCs (F‐PDLSCs) and normal PDLSCs (N‐PDLSCs) as determined by CCK‐8 assay. F‐PDLSCs and N‐PDLSCs isolated ex vivo proliferated at a similar rate for 1‐2 d, but F‐PDLSCs showed faster proliferation after 3 d. N = 6, *P < .05, **P < .01 vs N‐PDLSCs. B, Representative images and quantitative comparison of colony‐forming units‐fibroblastic (CFU‐F) of two different rPDLSCs. N = 6, *P < .05 vs N‐PDLSCs. C, Compared to N‐PDLSCs, the F‐PDLSCs showed a decreased capacity to form mineralized nodules, assessed by ARS staining and quantification. N = 5, ***P < .001 vs N‐PDLSCs. D, Oil Red O staining and quantification of two different rPDLSCs. F‐PDLSCs showed less accumulation of lipid‐rich vacuoles. N = 5, *P < .05 vs N‐PDLSCs. Scale bar: 400 μm. E, Representative images of crystal violet staining of RAW264.7 macrophages in Transwell assays. Conditional medium from F‐PDLSCs enhanced the migration of macrophages compared with the control. N = 6, **P < .01 vs N‐PDLSCs. Scale bar: 400 μm. F, Representative images of TRAP staining of osteoclasts among RAW264.7 macrophages co‐cultured with PDLSCs. Osteoclastic differentiation of RAW264.7 macrophages was significantly enhanced by force loading. N = 6, **P < .01 vs N‐PDLSCs. Scale bar: 200 μm. G, Relative mRNA levels of inflammation‐related genes. The mRNA levels of IL‐1β, TNF‐α, IL‐6 and MCP‐1 were upregulated in the F‐PDLSC group. ***P < .001 vs N‐PDLSCs. H, Western blot of Ki67. The protein level of Ki67 was upregulated in the force‐treated PDLSC group. *P < .05, **P < .01 vs N‐PDLSCs. Three independent assays were performed for each cell population

CCK‐8 assays revealed that proliferation of the force‐stimulated rPDLSCs (F‐PDLSCs) was stronger than that of normal PDLSCs (N‐PDLSCs), which was consistent with the phenomenon in vivo (Figure 2A). Both cell types proliferated slowly from 0 to 3 days. However, from 3 to 7 days, the F‐PDLSCs grew more rapidly than the N‐PDLSCs. Additionally, the F‐PDLSCs showed stronger colony formation ability compared with the N‐PDLSCs (Figure 2B). To evaluate their multi‐differentiation ability, cell populations at passage 2 were supplemented with osteoinductive and adipoinductive medium. After 3 weeks of osteogenic induction, the N‐PDLSCs produced dramatically more mineralized nodule (stained with Alizarin Red S) than the F‐PDLSCs (Figure 2C). Moreover, the F‐PDLSCs also suffered observable impairment of adipogenic differentiation, as shown by decreased accumulation of lipid‐rich vacuoles. Quantitative analysis showed that the number of lipid‐specific Oil Red O‐positive cells in F‐PDLSCs was less than that in N‐PDLSCs (Figure 2D). These data suggest that rPDLSCs showed greater proliferation and reduced differentiation ability following mechanical stimulation in vivo.

To evaluate the immunomodulatory function of the rPDLSCs, we performed a Transwell migration assay with F‐PDLSCs or N‐PDLSCs and RAW264.7 macrophages. We found that conditioned medium from the F‐PDLSCs evidently promoted the migration of macrophages compared with the N‐PDLSC group (Figure 2E). We further assessed paracrine impact on the osteoclast differentiation of F‐PDLSCs by using a cell‐to‐cell contact co‐culture system with rPDLSC and RAW264.7 macrophages. TRAP staining showed that the F‐PDLSCs enhanced osteoclast differentiation compared with the N‐PDLSC group (Figure 2F). In addition, the expression of proinflammatory factors in the rPDLSCs isolated ex vivo was evaluated. The mRNA levels of IL‐1β, TNF‐α, IL‐6 and MCP‐1 were increased in the F‐PDLSCs (Figure 2G). Moreover, the Ki67 protein level was upregulated after mechanical loading, which further confirmed the result of CCK‐8 assay (Figure 2H). Therefore, these results collectively suggest that positive paracrine effect of F‐PDLSCs, including macrophage migration and induction of osteoclast differentiation, might be attributed to enhanced proinflammatory factor expression in F‐PDLSCs. To sum up, mechanical stimuli in vivo might alter the MSC characteristics of rPDLSCs, which may contribute to bone remodelling and tooth movement.

### 3.3 TRPV4 channels are activated in the force‐stimulated rPDLSCs

**Figure 3:**
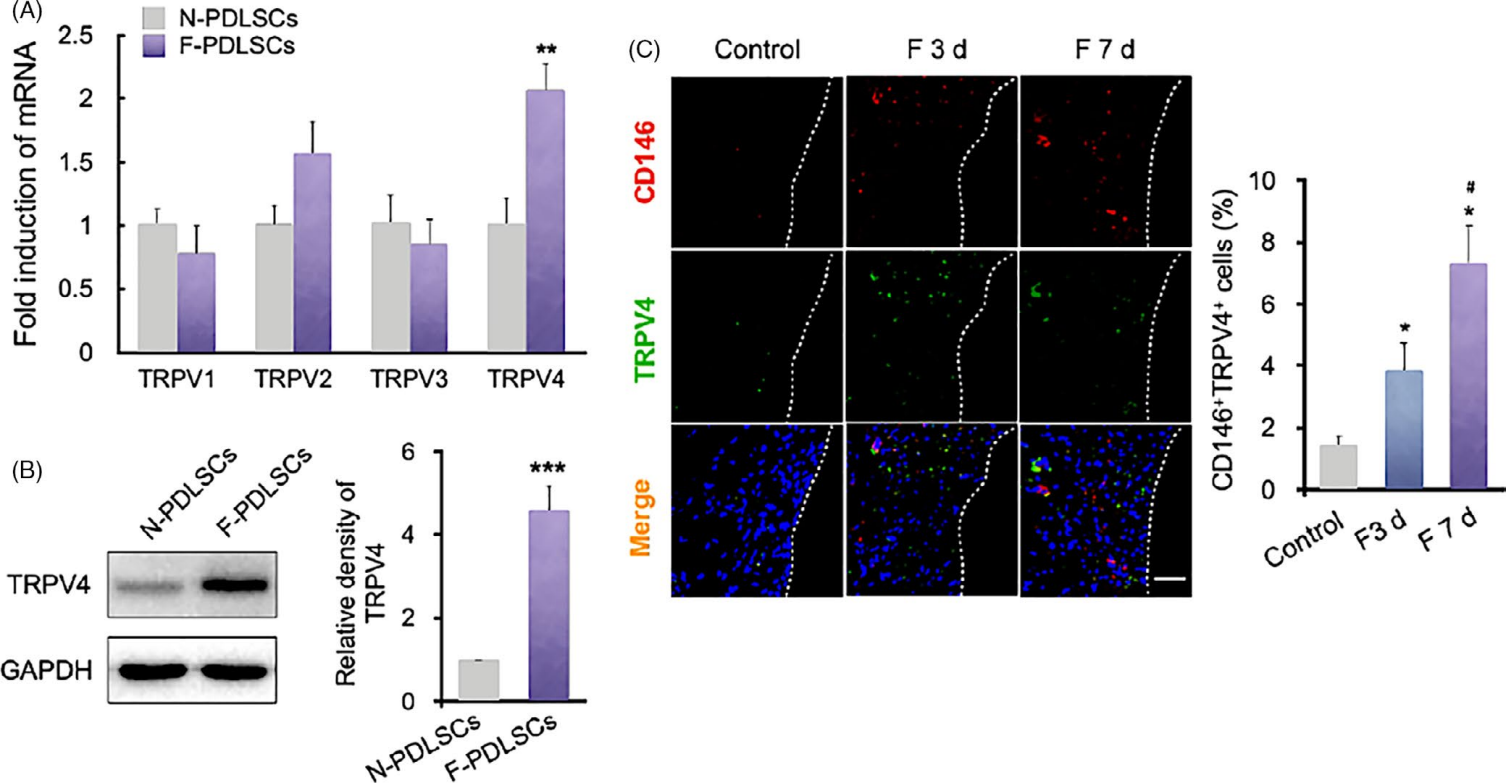
TRPV4 is present in F‐PDLSCs ex vivo. A, Relative mRNA levels of TRPV1‐4. TRPV1‐4 mRNAs were detected in rPDLSCs, while that of TRPV4 was increased in the F‐PDLSCs. **P < .01 vs N‐PDLSCs. B, Western blot of TRPV4 in rPDLSCs. The TRPV4 protein level in rPDLSCs was upregulated after force loading. ***P < .001 vs N‐PDLSCs. C, Representative immunofluorescence images and semi‐quantitative analysis of the compression side of distobuccal roots. The number of CD146^+^ (red) and TRPV4^+^ (green) PDLSCs was increased in F 3 d and F 7 d. N = 6, *P < .05 vs control, ^#^ *P* < .05 vs F 3 d. Scale bar: 100 μm. Data are means ± SD of three independent experiments

We next determined how mechanical force was perceived by rPDLSCs and translated into biological signals. Previous studies have demonstrated that TRPV channels are sensitive to microenvironment temperature, mechanical and chemical stimuli.^16^, ^25^ We therefore assayed the mRNA levels of TRPV channels in rPDLSCs. mRNA expression of TRPV1‐4 was detected in rPDLSCs, while that of TRPV4 was increased in the F‐PDLSCs (Figure 3A). The TRPV4 protein level in the F‐PDLSCs was also about fourfold than in N‐PDLSCs, consistent with the mRNA level (Figure 3B). To confirm the increased TRPV4 expression after mechanical loading in vivo, double immunostaining of TRPV4 and CD146 was performed during mechanical force‐mediated tooth movement. After force application, the proportion of CD146^+^TRPV4^+^ PDLSCs increased to 3.9 ± 0.9% at 3 days and 7.3 ± 1.3% at 7 days compared with the control group (Figure 3C). These results suggested that TRPV4, as a sensor of mechanical force, was activated in PDLSCs after mechanical stimulation, which may link the transduction of mechanical stimuli with the subsequent biological responses.

### 3.4 Inhibition of TRPV4 represses the biological characteristics of force‐stimulated rPDLSCs

**Figure 4:**
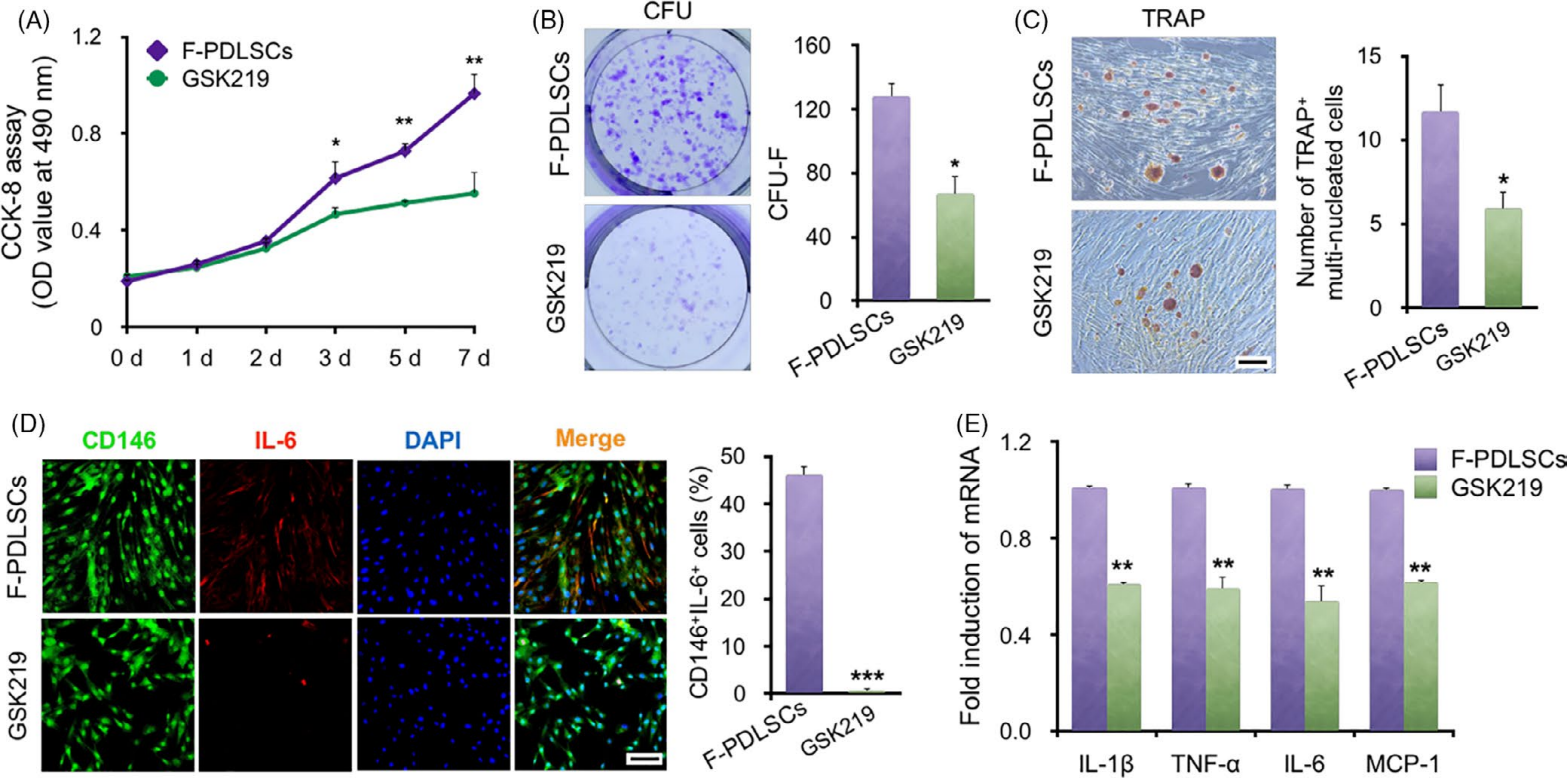
Inhibition of TRPV4 represses biological characteristics of F‐PDLSCs ex vivo. A, Growth curves of F‐PDLSCs and GSK219‐pre‐treated PDLSCs (GSK219). CCK‐8 assays showed that the promotion of proliferation after force loading was inhibited by the TRPV4 antagonist GSK219. N = 6, *P < .05, **P < .01 vs F‐PDLSCs. B, Representative images and quantitative comparison of CFU‐F of two different rPDLSCs. N = 6, *P < .05 vs F‐PDLSCs. C, Representative images of TRAP staining of osteoclasts among RAW264.7 macrophages co‐cultured with rPDLSCs. TRAP staining showed a significant decline in the number of TRAP^+^ osteoclasts among GSK219‐pre‐treated PDLSCs. N = 6, *P < .05 vs F‐PDLSCs. Scale bar: 200 μm. D, Representative immunofluorescence images of F‐PDLSCs and GSK219‐pre‐treated PDLSCs. The number of CD146 (green) and IL‐6 (red) double‐stained PDLSCs decreased after GSK219 treatment. N = 5, ***P < .001 vs F‐PDLSCs. Scale bar: 50 μm. E, Relative mRNA levels of inflammation‐related genes. The mRNA levels of IL‐1β, TNF‐α, IL‐6 and MCP‐1 were decreased in GSK219‐pre‐treated PDLSCs. Three independent assays were performed for each cell population

To explore whether TRPV4 participates in modulating PDLSC function under mechanical force, a small‐molecule antagonist, GSK2193874 (hereafter GSK219),^26^ was applied to functionally confirm the impact of TRPV4 on the F‐PDLSCs. CCK‐8 assays showed that the enhanced proliferation rate of F‐PDLSCs was inhibited by the TRPV4 antagonist GSK219 at 10 μmol/L (Figure 4A). The colony formation ability of the F‐PDLSCs was also impaired by GSK219 treatment (Figure 4B). In addition, TRAP staining results showed a significant decline in TRAP^+^ osteoclast differentiation in co‐culture of F‐PDLSCs and monocytes following treatment with GSK219 (Figure 4C). Immunofluorescence staining showed that IL‐6 expression in F‐PDLSCs was suppressed by GSK219 treatment (Figure 4D). Furthermore, the mRNA levels of IL‐1β, TNF‐α, IL‐6 and MCP‐1 were decreased in F‐PDLSCs after GSK219 treatment (Figure 4E). These results collectively suggested that force‐induced TRPV4 is involved in regulation of the proliferative capacity, colony‐forming ability of F‐PDLSCs, and mediates the paracrine effect on osteoclastic differentiation by secreting inflammation‐related genes.

### 3.5 Modulation of the NF‐κB ligand/osteoprotegerin ratio by TRPV4 in hPDLSCs under mechanical force in vitro

**Figure 5:**
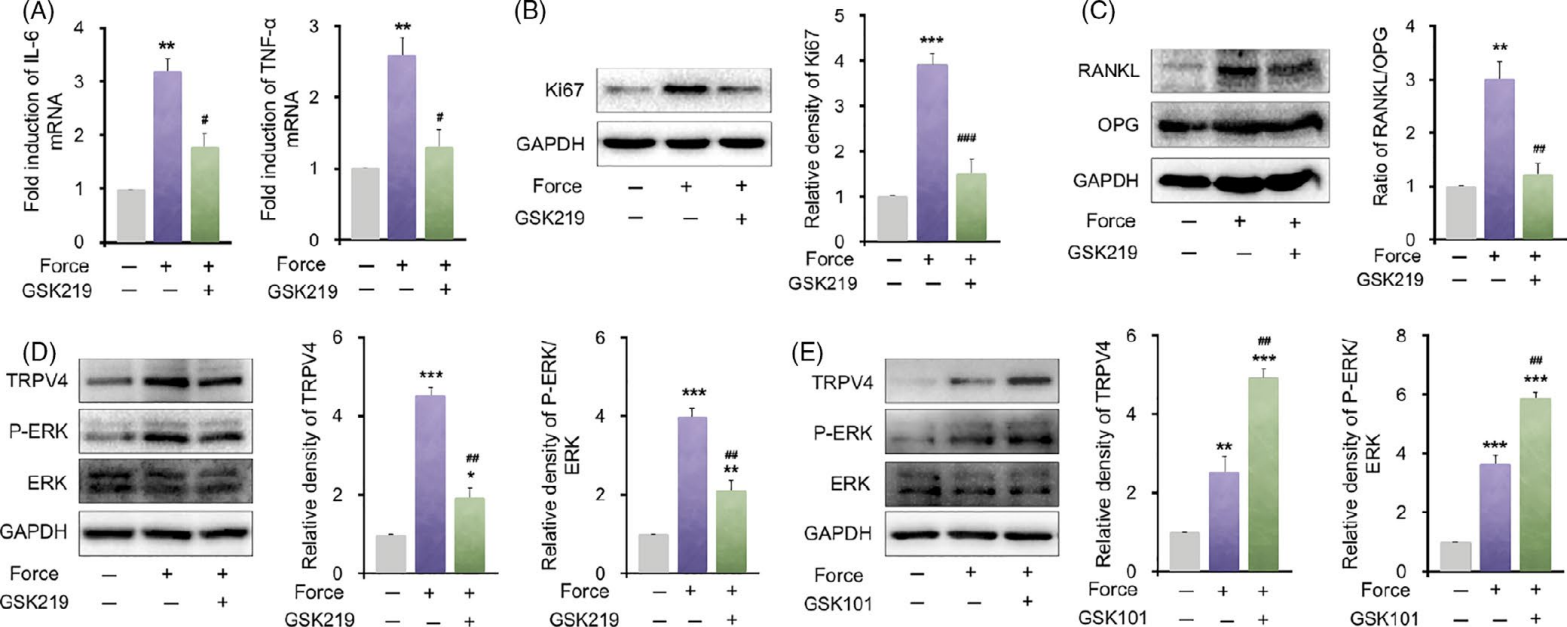
TRPV4 regulates force‐induced inflammation‐related gene expression and the receptor activator of nuclear factor‐κB ligand (RANKL)/osteoprotegerin (OPG) system in hPDLSCs via the ERK signalling pathway. A, Relative mRNA levels of inflammation‐related genes. The mRNA levels of IL‐6 and TNF‐α were upregulated in the force group and downregulated in the force + GSK219 group compared with the force group. B, Western blot and semi‐quantifications of Ki67 in hPDLSCs. The protein level of Ki67 was upregulated after mechanical force loading, which was mostly reversed by TRPV4 treatment. GAPDH served as an internal control for equal loading. C, GSK219 inhibition of TRPV4 decreased the force‐induced upregulation of the RANKL/OPG ratio. The protein levels of RANKL and OPG were determined in control PDLSCs or cells subjected to mechanical force with or without GSK219 treatment. D, Western blot and semi‐quantifications of TRPV4, phosphorylated ERK (P‐ERK), and total ERK (ERK) levels in hPDLSCs. The TRPV4 level and the proportion of P‐ERK/ERK were upregulated after mechanical force application and attenuated by the inhibition of TRPV4. E, Western blot and semi‐quantifications of TRPV4, P‐ERK and total ERK levels in hPDLSCs. The TRPV4 level and the proportion of P‐ERK/ERK were upregulated after mechanical force stimulation and further enhanced by a simultaneous treatment with GSK101. Data are means ± SD of three independent experiments. *P < .05, **P < .01, ***P < .001 vs control. ^#^ *P* < .05, ^##^ *P* < .01, ^###^ *P* < .001 vs force

TRPV4 was activated during bone remodelling in the process of tooth movement, and modulated the biological properties of force‐induced PDLSCs. Hence, we evaluated the mechanism by which TRPV4 expression in PDLSCs regulated osteoclastogenesis under mechanical force in vitro. Static compressive force‐treated hPDLSCs showed a dose‐dependent increase in the TRPV4 protein level (Figure S2). The mRNA levels of IL‐6 and TNF‐α in hPDLSCs were upregulated after compressive force loading in vitro and downregulated by GSK219 (Figure 5A). Consistently, Western blotting results showed that Ki67 protein level was upregulated after compressive force loading in vitro and downregulated by simultaneous treatment with GSK219 (Figure 5B and Figure S3). Moreover, the ratio of receptor activator of NF‐κB ligand/osteoprotegerin (RANKL/OPG), which is essential for osteoclast differentiation, was next assessed by Western blotting. Compressive force in vitro upregulated the RANKL protein level in hPDLSCs, which was reversed by simultaneous treatment with GSK219. By contrast, no significant change in the OPG level was detected. Therefore, the RANKL/OPG ratio increased after compressive force application, and this effect was partially blocked by simultaneous treatment with GSK219 (Figure 5C). These findings confirmed that force‐induced TRPV4 in hPDLSCs might modulate osteoclastic process by means of affecting RANKL/OPG system.

The signalling pathway by which TRPV4 in hPDLSCs regulated osteoclast generation was next investigated. ERK protein kinases can be activated by TRPV4 signalling. Additionally, ERK is involved in the transcriptional regulation of RANKL/OPG.^27^, ^28^ Therefore, we hypothesized that mechanical force‐induced upregulation of TRPV4 in hPDLSCs might increase the RANKL/OPG ratio by triggering the ERK signalling pathway. To this end, hPDLSCs were pre‐treated with a small‐molecule agonist or antagonist of TRPV4 before applying compressive force in vitro. Western blotting results showed that the compressive force caused rapid phosphorylation of ERK in hPDLSCs, which was partially attenuated by the additional intervention of TRPV4 inhibitor GSK219 (Figure 5D). Furthermore, addition of the TRPV4 agonist, GSK101 (10 nmol/L), to hPDLSCs enhanced induction of ERK phosphorylation after mechanical force stimulation (Figure 5E). These results suggest that force‐induced TRPV4 in hPDLSCs regulates osteoclast differentiation by affecting the RANKL/OPG system via ERK signalling.

## 4 DISCUSSION

Mechanical force plays an important role in tissue development and homoeostasis by modulating stem cell fate.^4^ As the main MSCs in the PDL, PDLSCs possess mechanosensitivity and responsiveness to mechanical stimulation both in vivo and in vitro,^29^, ^30^, ^31^ which endow PDLSCs with vital mission in maintaining periodontal tissue homoeostasis and guaranteeing alveolar bone remodelling.^8^, ^9^, ^10^ Moreover, it has been discovered that PDLSCs might mediate the inflammatory process and promote osteoclastogenesis under mechanical stimulation in vivo. In vitro, mechanical loading—including tension, compression and vibration—induces PDLSCs to express high levels of proinflammatory cytokines, chemokines, β‐2 adrenergic receptor and H_2_S.^11^, ^12^, ^13^, ^32^ However, the changes in the MSC characteristics of PDLSCs after the mechanical stimulus in vivo were unclear. Meaningfully, our study demonstrated that mechanical force in vivo also altered the characteristics of rat primary PDLSCs in terms of promoting their proliferation, proinflammatory cytokine secretion and influencing immunoregulation of macrophage migration and osteoclast differentiation in paracrine manner, but suppressed their differentiation ability. On the whole, these results indicate the changes in the MSC characteristics of PDLSCs were induced by mechanical force in vivo.

Mesenchymal stem cells are sensitive to mechanical force. Until now, various mechanosensors have been proposed to be involved in modulating MSC differentiation under mechanical stimuli in vitro, including the mobilization of second messengers, ion channels, the cytoskeleton, primary cilia, membrane proteins such as integrins, or through changes in cellular structure.^14^, ^15^ However, the mechanotransduction mechanism of PDLSCs, the main MSCs in the mechanical sensor of periodontal tissues, is unclear. Ca^2+^ influx in PDLSCs has been reported following mechanical stimuli.^21^ Previous studies have shown that the level of cytoskeletal remodelling influenced the mechanically driven osteogenic differentiation of PDLSCs.^33^ A piezo channel has also been proven to sense mechanical signals and regulate stem cell behaviours^34^; however, it is reportedly not associated with ultrasound‐related signal‐stimulated PDLSC proliferation.^35^ In this study, the expression of TRPV4, a calcium channel, was increased in PDLSCs under mechanical stimulation in vivo. This suggests that TRPV4 plays an important role in the transduction of mechanical stimuli in PDLSCs and may mediate subsequent biological responses.

TRPV4 is a calcium channel involved in the sensation of different stimuli in various cells and tissues.^18^, ^19^, ^36^ TRPV4 activation is associated with the inflammatory response and promotes proinflammatory cytokine release by various types of tissues and cells. Moreover, mutations in TRPV4 are linked to inherited disorders of bone metabolism.^18^ MSCs isolated from TRPV4‐knockout mice demonstrated an impaired osteogenic potential.^19^ In this study, we proved that TRPV4 mediated the mechanical response of PDLSCs both in vivo and in vitro. Firstly, the expression of TRPV4 was upregulated in PDLSCs under mechanical stimulation in vivo. Next, inhibition of TRPV4 in force‐loaded PDLSCs isolated ex vivo decreased their proliferation, and secretion of inflammation‐related cytokines, which then restrains osteoclastic differentiation. Consistently, TRPV4 activation mediated hPDLSCs biological responses to mechanical force in vitro, which were restricted by a simultaneous treatment with TRPV4 inhibitor GSK219. Overall, above results indicate that compressive force increased TRPV4 expression and function, and thereby triggering subsequent behaviours, which can be partially blocked by TRPV4 deactivation.

Periodontal ligament stem cells modulate alveolar bone remodelling under mechanical stimuli by inducing bone formation on the tension side and bone resorption on the compression side of periodontal tissues.^37^ Zhang et al^38^ found that compressive force activated the Wnt/β‐catenin pathway in human PDLSCs. Our previous studies showed that PDLSCs highly express β‐2 adrenergic receptor and produce a high level of H_2_S under a static compression stimulus, both of which could promote osteoclastic differentiation by increasing the RANKL/OPG ratio.^21^, ^29^ In addition, PDLSCs also induce polarization of inflammatory M1 macrophages, thereby contributing to osteoclastogenesis. In this study, the ERK protein kinase signalling pathway, which is essential for osteoclastogenesis, was explored.^39^, ^40^ The static compressive force induced the phosphorylation of ERK in PDLSCs, which was partially rescued by treatment with the TRPV4 inhibitor and enhanced by treatment with the TRPV4 agonist. Furthermore, alteration of RANKL/OPG ratio keeps consistent with phosphorylation of ERK under corresponding intervention. These data suggest that mechanical force‐induced TRPV4 activation in PDLSCs might promote osteoclastogenesis by regulating the RANKL/OPG ratio via the ERK signal pathway. Nevertheless, PDLSCs on the tension side may response differently, comparing to those on the compression side.^41^ The reaction of PDLSCs to tensile strain during OTM would be investigated in future. In addition, further studies would be performed on TRPV4 knockout mice to confirm the functional role of TRPV4 on alveolar bone remodelling and tooth movement.

## 5 CONCLUSION

**Figure 6:**
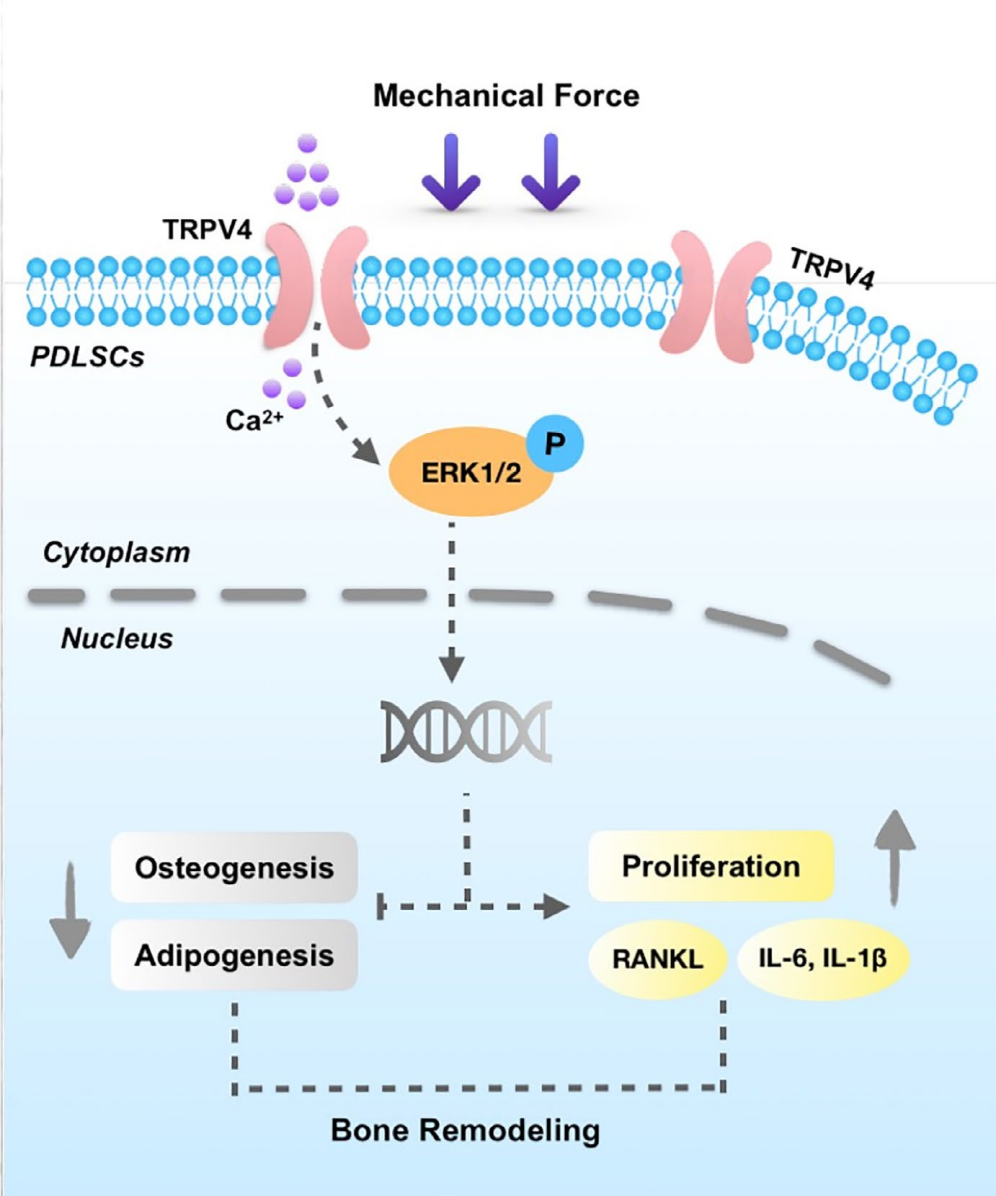
The activation of TRPV4 in PDLSCs under mechanical force contributes to the changes in their biological properties and modulates bone remodelling during tooth movement

In summary, we show here that the activation of TRPV4 in PDLSCs under mechanical force contributes to the changes in their biological properties including clonogenicity, proliferation, multipotent differentiation, and immunoregulation and modulates bone remodelling during tooth movement (Figure 6). These results suggest a critical role for PDLSCs in mechanical force‐induced bone remodelling and indicate the importance of TRPV4 in regulating PDLSC function and mediating bone remodelling under mechanical force. The findings also imply that targeting TRPV4 might benefit mechanical force‐induced bone remodelling and tooth movement.

## CONFLICT OF INTEREST

The authors declare that they have no competing interests.

## AUTHORS' CONTRIBUTIONS

SJ and DH performed the experiments, collected and analysed the data, and wrote the manuscript. YW, TZ, HY and YZ contributed to collection and assembly of data. XL and LZ performed the experiments. DH and YL contributed to overall design of the study, critically editing the manuscript. All authors reviewed and approved the manuscript.

## ETHICAL APPROVAL

The animal experimental protocols were approved by the Institutional Animal Care and Use Committee of Peking University (LA2013‐92). Permission to obtain extracted teeth for human PDLSCs was provided by the Ethics Committee of Peking University (PKUSSIRB‐201311103).

## CONSENT FOR PUBLICATION

Not applicable.

## Supporting information

Supplementary Material

## Data Availability

The data sets used and/or analysed during the current study are available from the corresponding author on reasonable request.
