# Supplementary material for: Mechanical force modulates periodontal ligament stem cell characteristics during bone remodelling via TRPV4
Source: Cell Prolif. 2020 Sep 22;53(10):e12912. doi: 10.1111/cpr.12912 (PMC7574874; doi:10.1111/cpr.12912)
Supplement: Supplementary file 1 — Supplementary Material [file CPR-53-e12912-s001.docx]

**Supporting Information**

**Mechanical force modulates** **periodontal ligament stem cell characteristics during bone remodeling via TRPV4**

Shan-Shan Jin,^a†^ Dan-Qing He, ^a†^ Yu Wang,^a^ Ting Zhang,^a^ Hua-Jie Yu,^b^ Zi-Xin Li,^a^ Li-Sha Zhu,^a^ Yan-Heng Zhou, ^a^ Yan Liu ^a*^

^a^ Laboratory of Biomimetic Nanomaterials, Department of Orthodontics, Peking University School and Hospital of Stomatology, National Engineering Laboratory for Digital and Material Technology of Stomatology, Beijing Key Laboratory of Digital Stomatology, Beijing 100081, China.

^b^ Peking University Hospital of Stomatology, Fourth Division, Beijing 100025, China.


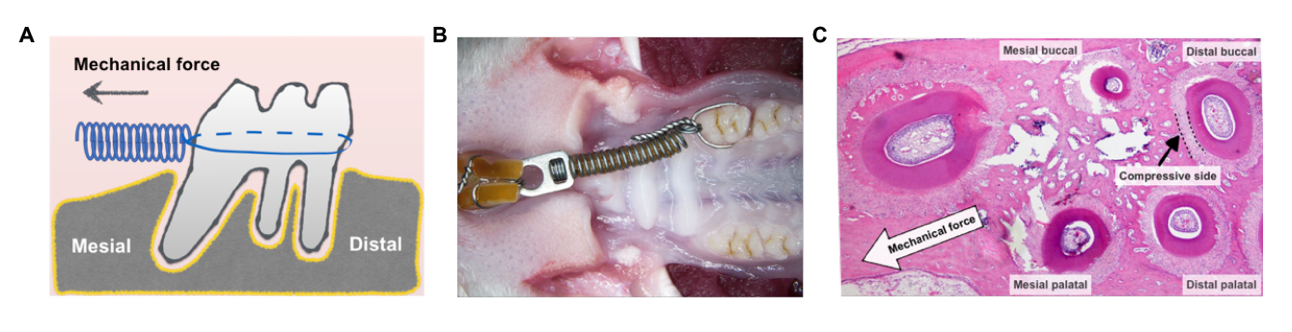
**Figure S1.** Schematic illustration. (A) Schematic of mechanical loading. (B) The occlusal view of mechanical loading. (C) Schematic illustration showing the area of investigation (dotted line) in the mesial aspect of the distal buccal root of the first molar.


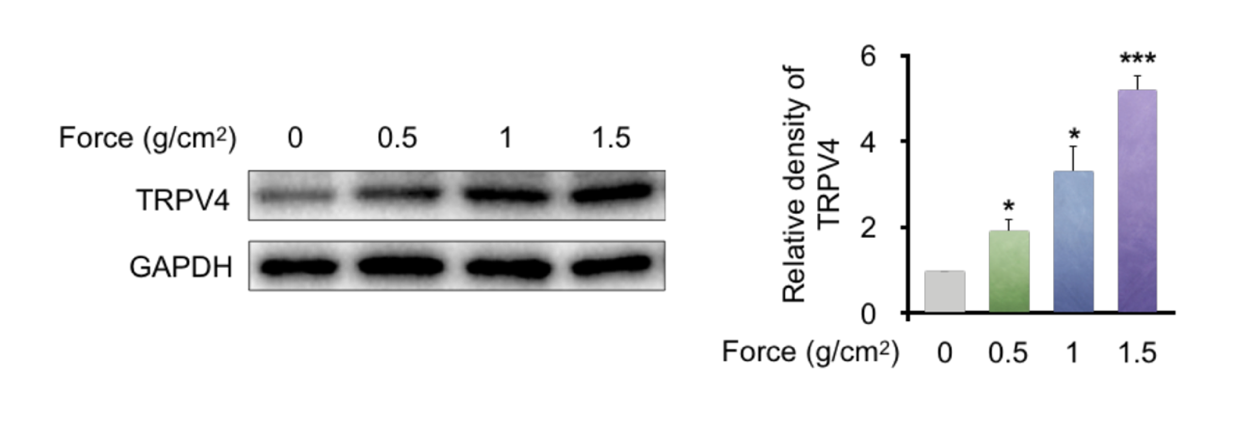


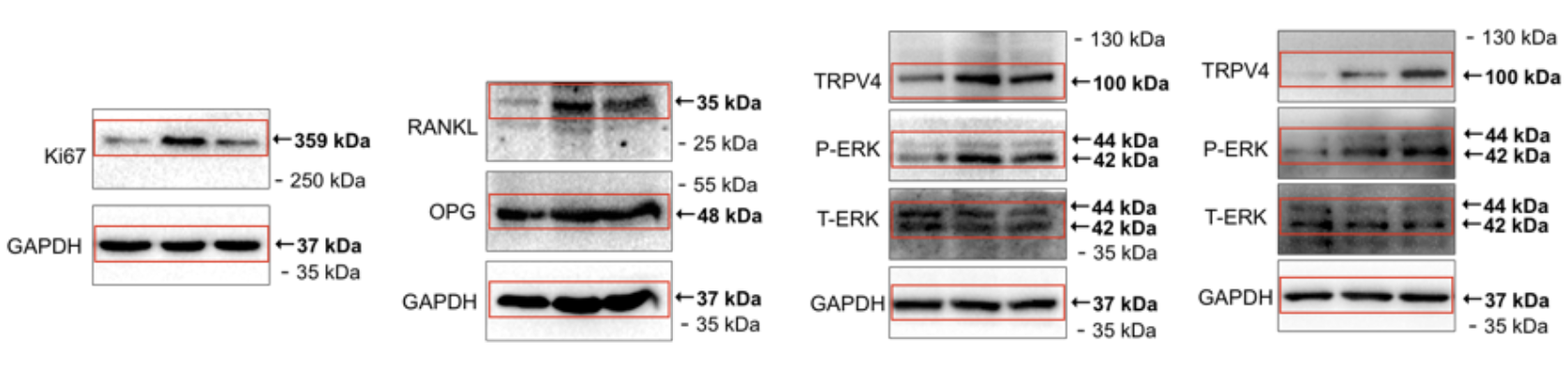
**Figure S2.** Western blot of TRPV4 protein expression. TRPV4 expression in PDLSCs was upregulated with increasing force-treated intensity for 12 h. *P < 0.05, ***P < 0.001 *versus* 0 g/cm^2^ group.

**Figure S3.** Full-size images of the Western blotting in Figure 5.

**Supplementary Table 1.** List of primers used in the study.

| **Primer** | Sense primer (5’-3’) | Antisense primer (5’-3’) |
| --- | --- | --- |
| Rat | | |
| β-actin | TGACAGGATGCAGAAGGAGA | TAGAGCCACCAATCCACACA |
| IL-1β | CACCTCTCAAGCAGAGCACAG | GGGTTCCATGGTGAAGTCAAC |
| TNF-α | CCAGGTTCTCTTCAAGGGACAA | CTCCTGGTATGAAATGGCAAATC |
| IL-6 | CCAAGACCATCCAACTCATCTTG | CACAGTGAGGAATGTCCACAAAC |
| MCP-1 | CAGCCAGATGCAATCAATGCC | TGGAATCCTGAACCCACTTCT |
| TPRV1 | GCCGCTGAACCGACTC | CCCATCTGCTGGAAAC |
| TPRV2 | CGCCATTGAGAAGAGGAGTC | GCTTACCACATCCCACTGCT |
| TPRV3 | GCGTGGAGGAGTTGGTAGAG | CTCTGTGTACTCGGCGTTGA |
| TPRV4 | CAGGTGGGGAGGCTTTT | GCGGCTGCTTCTCTATG |
| Human | | |
| GAPDH | ATGGGGAAGGTGAAGGTCG | GGGGTCATTGATGGCAACAATA |
| TNF-α | CCTCTCTCTAATCAGCCCTCTG | GAGGACCTGGGAGTAGATGAG |
| IL-6 | CGGTCCAGTTGCCTTCT | GCCAGTGCCTCTTTGCT |
